# Fish diversity patterns along coastal habitats of the southeastern Galapagos archipelago and their relationship with environmental variables

**DOI:** 10.1038/s41598-022-07601-w

**Published:** 2022-03-04

**Authors:** Marjorie Riofrío-Lazo, Manuel J. Zetina-Rejón, Leandro Vaca-Pita, Juan Carlos Murillo-Posada, Diego Páez-Rosas

**Affiliations:** 1 Universidad San Francisco de Quito and Galapagos Science Center, Isla San Cristóbal, Galápagos, Ecuador; 2 Instituto Politécnico Nacional, Centro Interdisciplinario de Ciencias Marinas (CICIMAR-IPN), La Paz, Baja California Sur México; 3 Guía Naturalista en Patrimonio Turístico del Parque Nacional Galápagos, Islas Galápagos, San Cristóbal, Ecuador; 4 Pontificia Universidad Católica del Ecuador-Sede Manabí, Facultad de Biología, Bahía de Caráquez, Ecuador; 5 Dirección del Parque Nacional Galápagos, Unidad Técnica Operativa San Cristóbal, Isla San Cristóbal, Galápagos, Ecuador

**Keywords:** Ecology, Biodiversity, Ichthyology, Community ecology

## Abstract

Coastal habitats are essential for ecological processes and provide important ecosystem services. The Galapagos archipelago has a wide diversity of ichthyofauna which preservation guarantees the functioning of the marine ecosystem. In this study, we used ecological and taxonomic indices as well as multivariate analysis to identify spatiotemporal changes in fish community structure in coastal habitats of San Cristóbal Island in the southeastern Galapagos archipelago. We analyzed how the patterns of variability were related to the abiotic conditions (substrate, sea temperature and depth) of each habitat. Nine sites affected by anthropogenic influence (fishing and tourism) representing different habitats/substrates were sampled. Underwater surveys were conducted during the warm and cold seasons in 2010 and 2011 at transects that varied in depth according to site. Artificial habitat, followed by coral and rocky habitats, had the highest diversity, evenness, and taxonomic distinctness, while mangrove habitats had the lowest values. This was related to the habitat complexity and possible anthropogenic influences. While the diversity patterns were more strongly related to the type of substrate, followed by the combination of substrate and depth, and the sea temperature had less influence. These findings were related to the ecological traits of the fish communities and their mobility between habitats. Temporal changes in fish community diversity and composition were not detected at all sites, suggesting that these species have high fidelity to their habitats and a high environmental tolerance that allows them to persist in their habitats despite strong changes in sea temperature on the Galapagos archipelago.

## Introduction

The Galapagos archipelago has an extensive marine zone that represents a unique hotspot of marine species diversity; these species have colonized this region because of the presence of characteristic marine currents^1–3^. This situation has resulted in diverse marine fish fauna^4,5^. There are 128 families of fishes that have been reported in the Galapagos, of which approximately 75 species are endemic^6,7^. Approximately 444 fish species have been described^8^, and their distributions are associated with multiple factors, such as resource availability, substrate topography, marine currents, and species behavior^9–11^.

The Equatorial Undercurrent and the South Equatorial current are important in the Galapagos as they supply a macronutrients influx and transport the larvae of different species^6,12^. These currents influence the levels of marine productivity, creating a series of regions within the archipelago^13,14^, which are distinguished by a mix of Panamanian, Peruvian, Indo-Pacific, and endemic fish species^2,11^. The waters of Equatorial Undercurrent are the most nutrient-rich and generate continuous upwellings, mainly in the western region, which contribute to phytoplankton blooms, leading to an increased abundance and diversity of species^13,15^.

Productive habitats are also present near the coasts of southeastern islands (Floreana, San Cristóbal, Española), with varying persistence over time^15,16^. The zones surrounding the coastlines of islands, inlets, and rocks are relevant habitats in the archipelago where important ecological processes take place^17,18^. The coastal habitats, such as rocky reefs, mangroves, coral zones, and sand beaches, on the archipelago are productive areas that supply important ecosystem functions, such as feeding, protection and reproduction areas. A variety of species inhabit these areas through some or all phases of their life span^2,12,19–21^. Therefore, these habitats are essential as feeding areas, nurseries, and spawning areas and for fish migration with commercial and ecological relevance^22–25^. Fish frequently depend on distinct habitats with different structural complexities and substrate composition through their life span and visit them seasonally^22^. Habitats are thus linked through species migration^26^.

The preservation of marine biodiversity guarantees the functioning of ecosystems^27^; therefore, the diminished biodiversity is particularly worrying because it is a difficult process to manage. Some ecological indices have been developed to evaluate the biodiversity of ecosystems, being the indices of diversity, evenness and richness those that are commonly used to compare fish community structures^25,28,29^. Multivariate analysis is an alternative tool for understanding spatiotemporal changes in biodiversity in different communities^30^. Since the number of species recorded in a site is highly related to sampling effort, these approaches evaluate variations in the taxonomic relatedness between species that could be linked to functional diversity^31,32^. Hence, they are used to compare spatiotemporal distributions of species and possible degraded areas^33–35^.

Despite the high ecological importance of the coastal habitats in the Galapagos Islands^17,18^, there is a gap regarding the taxonomic diversity evaluation of the fish communities and variability patterns among habitats, which is relevant from the ecosystem management perspective. The zoning of the Galapagos Marine Reserve is focused on protecting marine ecosystems and their biodiversity as well as regulating human activities, such as tourism and artisanal fishing within the archipelago^36,37^. However, the zoning system, established in 2000, is still considered preliminary as it does not adequately represent the conservation needs around the marine reserve. Therefore, more data on species diversity and their distribution in the marine ecosystem are required for reconfiguring these zones^17,18^.

The fish communities’ composition of the Galapagos is related to its geographical location and the influence of currents and water temperature that generate a remarkable environmental diversity^38^. Factors influencing patterns of variation in fish diversity among different coastal habitat types in the same region of the archipelago have not been thoroughly studied. However, it has been reported that the depth and habitat complexity, described by roughness and number of cavities, determine the structure of fish populations on rocky bottoms in the Galapagos Islands^39^. Furthermore, temperature gradients and the concentration of nutrients in the water (related to anthropogenic influence) likely influence the structure of fish assemblages in rocky habitats^40^. While, in mangrove habitats, the islands' isolation and their location in a convergence zone could influence the composition of fish communities between regions of the Galapagos^25^.

In this study, we compare the fish communities in coastal habitats (coral, rocky, mangrove, oceanic, and artificial) on San Cristóbal Island in the southeastern region of the Galapagos archipelago to determine possible differences in taxonomic diversity and composition among habitats by using ecological and taxonomic indices and multivariate analysis. We assessed which habitats show the highest taxonomic diversity and analyzed how the patterns of diversity variability relate to the abiotic conditions (substrate, depth and temperature) of each habitat. We hypothesized that in a same region of the archipelago, habitat complexity and depth are the variables more influencing fish communities' structure and diversity. While temporally, the seasonal sea temperature could determine the fish diversity patterns in coastal habitats.

## Methods

### Study area

**Figure 1:**
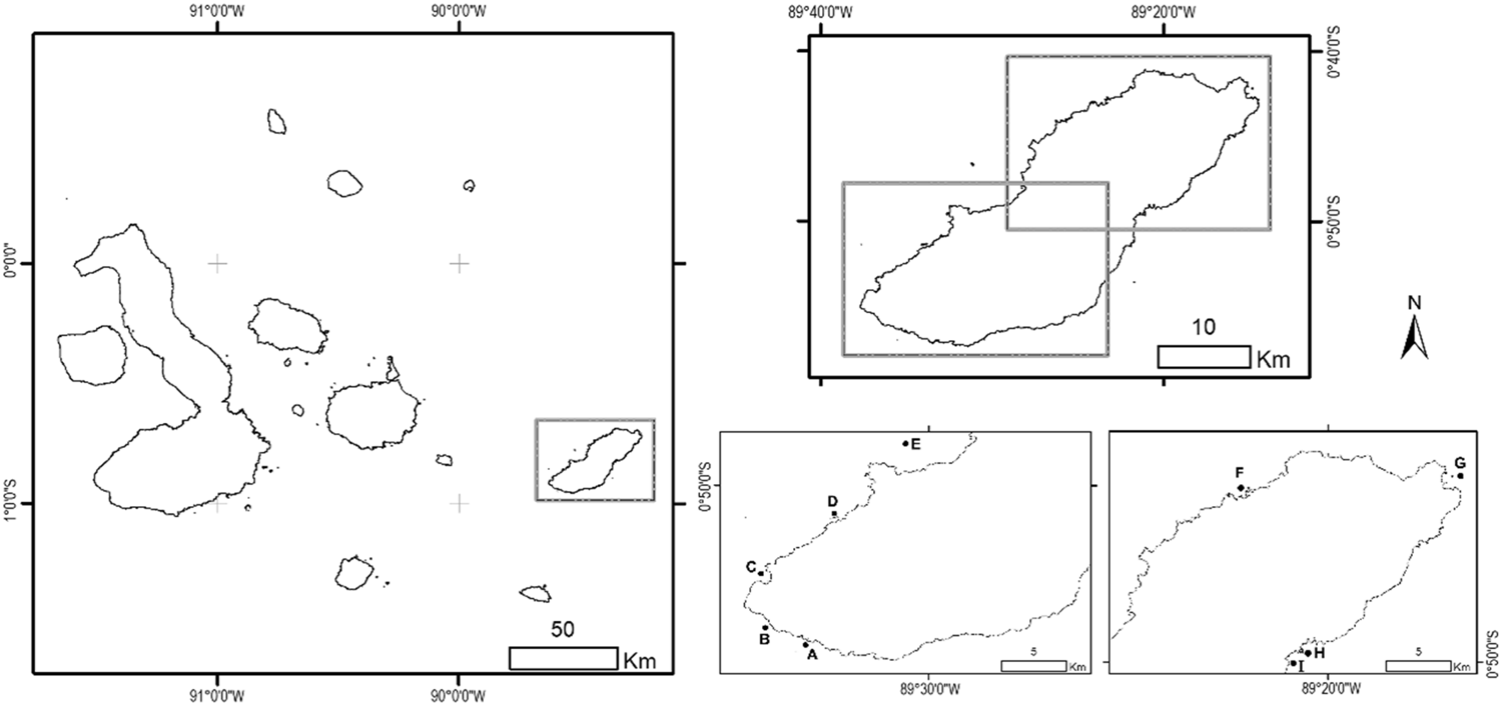
Study area showing the sampling sites (black dots) on the shelf of San Cristóbal Island in the southeastern Galapagos archipelago. (A) Las Negritas (0° 56′ 29.874″ S, 89° 35′ 07.84″ W), (B) La Lobería (0° 55′ 47.25″ S, 89° 36′ 45.65″ W), (C) Karahua (0° 53′ 43.34″ S, 89° 37′ 23.38″ W), (D) Isla Lobos (0° 51′ 23.01″ S, 89° 33′ 55.49″ W), (E) León Dormido (0° 46′ 42.51″ S, 89° 31′ 13.12″ W), (F) La Tortuga (0° 43′ 8.28″ S, 89° 23′ 29.99″ W), (G) Punta Pitt (0° 41′ 58.99″ S, 89° 14′ 42.24″ W), (H) Rosa Blanca-Coral (0° 49′ 43.60″ S, 89° 21′ 14.25″ W) and (I) Rosa Blanca-Mangrove (0° 49′ 51.25″ S, 89° 21′ 41.79″ W). The map was created using ArcGIS 10.5.1 (ESRI, https://www.esri.com).

The Galapagos Islands are situated approximately 1000 km from mainland Ecuador (Fig. 1). This archipelago has 15 major islands and is located within an upwelling system because of the convergence of several oceanic currents^13^. These currents show variations in their strengths throughout the year which result in two different seasons: a warm season (January to May) and a cold season (June to December) with temperatures higher than 25 °C, and between 18 and 24 °C, respectively^41^. This seasonality affects the sea surface temperature around the islands, ranging up to 8 °C between seasons^41^.

We carried out this research in coastal habitats of San Cristóbal Island, which is at the eastern end of the Galapagos archipelago (Fig. 1). This island has a shoreline perimeter of approximately 159 km and the subtidal zone can extend to 3 km from the coast, reaching depths of ~ 50 m^42^. More than 90% of the wide shelf is covered by a rocky reef habitat, which is complemented by few patches of mangrove areas and coral reefs^2,43^. These characteristics make this site a primary habitat for many fish species during their ontogenetic development, depending on their feeding, growth, reproduction and protection needs^8^. Nine sampling sites distributed throughout the study area were selected according to the type of habitat (Fig. 1). We refer to the habitat as the most dominant feature accountable for the environment structural complexity^44^, which can originate both from geological structures (e.g., rocky bottoms) or vegetation (e.g., mangroves).

Rocky habitat is characterized by an irregular sea bottom formed by lava rocks with pronounced rocky elevations producing a variety of caves and fissures. The Isla Lobos islet, Las Negritas and La Lobería represent rocky habitat. Isla Lobos islet is located northwest of the island 0.30 km from the coast; the outer part has an extensive rocky sea bottom with sand patches. Las Negritas, to the southwest of the island, has a very irregular sea bottom, mainly rocky with sand patches, small vegetated areas, and the presence of a few coral colonies from the *Pavona* genera. Finally, La Lobería, in the southwest of the island, has a wide coral beach and a sea bottom of rocky reefs.

The coral habitat presented an extensive submerged wall with numerous coral colonies of different species, including those of the *Pavona* and *Pocillopora* genera. Punta Pitt and Rosa Blanca represent coral habitat. Punta Pitt is a bay located north of the island; the outer part has a sea bottom with a wide coral and algae covering and some rocky formations. To the island's east, Rosa Blanca is a semi-closed coastal area whose outer part of greater depth (about 11.5 m) presents a sea bottom with numerous coral communities accompanied by rocks and sand patches.

The mangrove habitat was in shallow waters (up to 3 m of depth) distinguished by the presence of red mangroves (*Rhizophora mangle* L.) and a soft sea bottom mainly consisting of sand. The inshore zone in Rosa Blanca and La Tortuga represent mangrove habitat. At Rosa Blanca, the sea bottom is primarily sandy with few rocky areas. About 95% of the bay is covered by red mangrove trees and 5% by black mangrove (*Avicennia germinans* L.). La Tortuga, located to the island's northwest, is a coastal area surrounded by red mangroves and presents a sandy-rocky sea bottom.

The oceanic habitat corresponded to open waters of less than 30 m in depth that were not directly beside the coast but that had an extensive submerged wall composed of compacted volcanic ash. León Dormido (kicker rock) represents oceanic habitat; it is formed by two eroded volcanic tuff rocks located northwest of the island 5 km from the coast. Finally, the artificial habitat was represented by manmade structures constructed of hard substrates. Karahua (sunken ship) represents artificial habitat. This shipwreck locates to the east of the island 0.66 km from the coast, a large reef has formed around the ship's hull, and the sea bottom around it is of a sandy-rocky type.

### Data collection

Data were collected during months representative of the warm and cold seasons in 2010 and 2011 by underwater visual surveys using SCUBA diving and snorkeling. All sites were not sampled simultaneously but the number of transects sampled per season was similar among sites (Table 1). Censuses were carried out in a total of 180 transects (20 transects at each site) that varied in depth according to site. Dives were usually made at depths of 5 to 12 m in Isla Lobos, Las Negritas, La Lobería, Punta Pitt and Rosa Blanca-coral. The deepest dives (12 to 16 m) were made in León Dormido and Karahua, while shallow dives (1 to 3 m) were made in La Tortuga and the Rosa Blanca-mangrove. The censuses were conducted by a team of three divers early in the morning (08:00 to 10:00) when the exterior lighting favors the identification of species. The transects were linear (2 m wide by 50 m long) and were located at two meters in height, measured from the sea bottom towards the diver. At the León Dormido site, the transects were located at the submerged wall. At mangrove sites, transects were located next to the mangrove fringe to record fish up to 1 m inside the mangrove roots. Two divers moved forward, identifying and counting the species that were within two meters of their perspective (on both left and right sides of the transect). The third diver took pictures of all identified fishes and recorded sea bottom temperature and depth data with a dive computer. This methodology is commonly applied for ichthyofaunal ecological monitoring, as it is a noninvasive and nondestructive technique for gathering data on fish assemblages for further estimates of the density of underwater species^2,38,45,46^. Specialized identification keys^6^ were used to identify fish at species level and later corroborated by photo identification based on^47^ criteria. Since not all sites were sampled during the same year and the same months, we grouped the data into warm and cold seasons, and analysis was performed as follows.

**Table 1:** Samplings conducted at study sites during the warm and cold seasons. Dives depth (m) minimum and maximum values, number of transects surveyed, sampling dates, and sea temperature (°C) mean values (minimum and maximum in parenthesis) recorded per season and site.

Sampling site-substrate	Depth	Warm season			Cold season		
		Transects	Sampling date	Sea temperature	Transects	Sampling date	Sea temperature
Punta Pitt-Coral	5.3–10.4	10	30 January, 5 March, 16 April and 21 May 2010	28.30 (25.00–30.50)	10	18 June, 21 July, 4 August and 2 November 2010	19.65 (18.00–22.00)
Rosa Blanca-Coral	3.6–11.5	10	29 March, 1 May and 28 May 2010	27.20 (23.80–30.00)	10	1 July, 14 August and 1 September 2010	21.25 (20.00–22.70)
Rosa Blanca-Mangrove	1.5–3	10	5 March, 1 May and 28 May 2010	28.15 (25.50–30.50)	10	1 July, 14 August and 1 September 2010	24.30 (23.80–25.00)
Negritas-Rocky	4.5–14	10	9 March, 29 March and 1 May 2010	28.01 (25.50–30.50)	10	18 June, 4 August and 19 August 2010	20.04 (16.60–21.60)
La Loberia-Rocky	2–6	10	13 January, 13 March and 30 April 2011	22.00 (21.53–23.18)	10	5 September, 21 October and 19 November 2011	19.00 (17.51–21.24)
Karahua-Artificial	10–15	10	15 February, 22 March, 30 April and 10 May 2011	22.68 (22.00–23.00)	10	9 June, 11 August, 3 September, 21 October and 19 November 2011	18.72 (17.00–21.00)
Isla Lobos-Rocky	5.5–13	10	20 February, 16 April and 31 May 2010	26.26 (22.70–30.50)	10	26 June, 24 July and 19 August 2010	20.07 (18.90–21.60)
León dormido-Oceanic	12–16	10	26 March, 16 April and 28 May 2011	20.56 (20.00–21.00)	10	9 June, 11 August and 8 September 2011	19.00 (18.03–20.86)
La Tortuga-Mangrove	1–3	10	17 March, 10 April and 21 May 2010	28.12 (25.50–30.00)	10	26 June, 24 July, 19 August and 19 November 2010	20.77 (19.00–22.70)

### Data analysis

The numeric abundance was standardized to density by dividing the number of individuals by the number of transects surveyed in each site. We compared the fish community by site and substrate (i.e., rocky, coral, mangrove, oceanic and artificial) during the warm and cold seasons. For comparison purposes with other studies, we assessed the fish community with the most common related diversity indices in fish ecology, Shannon’s diversity index (H′), Pielou’s evenness (J′), the average taxonomic distinctness (Δ+), and the variation in taxonomic distinctness (Λ+), which estimates the taxonomic tree asymmetry^32^. For this, we used the following equations:

$${H}^{^{\prime}}=-{\sum }_{i}{p}_{i}\mathrm{ln}{p}_{i},$$

$$J^{\prime} = H^{\prime}/H_{max} ,$$

$$\Delta^{ + } = 2\frac{{\sum \mathop \sum \nolimits_{i < j} \omega_{ij} }}{{S\left( {S - 1} \right)}},$$

$$\Lambda^{ + } = 2\frac{{\sum \mathop \sum \nolimits_{i < j} \left( {\omega_{ij} - \Delta^{ + } } \right)^{2} }}{{S\left( {S - 1} \right)}},$$

where *pi* is the relative abundance of each species, corresponding to the proportion of individuals of a species concerning the total individuals in the community; *H*_*max*_ represents the highest Shannon–Wiener diversity possible value, reached when the species have equal abundances; *S* is the number of species observed in the sample, and *w*_ij_ is the weight given to the branch length between species pair *i* and *j* in the hierarchical classification. For the taxonomic classification, we used the data retrieved from FishBase (https://www.fishbase.de/) and considered six hierarchies (class, order, family, genus and species) for each taxon. The average taxonomic distinctness (∆+) estimation was based on presence/absence data at each site. In addition, we estimated ∆+ and Λ+ from 1000 simulated subsamples with different numbers of species. We used those estimations to generate 95% probability funnels that were then plotted versus the observed values. In this way, we compared the observed values of ∆+ and Λ+ against the expected values based on random samples. Because the sampling effort does not influence the values of ∆+ and Λ+ , both indicators can be used for comparison with future research^30,48^.

In order to identify patterns in the fish community at the sampling sites, a non-metric multidimensional scaling (MDS) analysis was used. MDS is a useful technique to visualize the similarities of data of the same type (e.g., abundance of species). The MDS was carried out by a similarity matrix of Bray–Curtis estimated from a square-root-transformed fish abundance matrix. In the MDS, sampling sites are represented in two-dimensional space, thus the relative distances among sites are in the same rank order. Later, we used an analysis of similarity (ANOSIM) to assess if the community associations do not differ spatially^49^. Additionally, we used a BIOENV test employing Spearman’s rank correlation coefficients to identify which abiotic variables are associated to the fish community structure^30,50^. For each sampling site, biotic data consisted of the species abundance and abiotic data were based on a categorical variable for substrate and two continuous variables, depth, and temperature. We used the same biotic matrix employed in the MDS and ANOSIM. We used the similarity percentages (SIMPER) algorithm to determine the species responsible for the differences among groups detected by the MDS^49^. This approach is based on decomposing dissimilarities for measuring the individual species contribution to the overall dissimilarity. Species that accounted for at least 70% of the dissimilarities were identified as responsible for the differences between groups.

To assess statistical differences in the temperature, species richness, diversity of the fish community, evenness, and average taxonomic distinctness values among sites, we performed a Kruskal–Wallis test. We test differences between seasons by a Wilcoxon paired test. The statistical significance was based on the “P-value” at the 0.05 level.

## Results

We identified an overall of 43,318 individuals from 75 fish species (Supplementary Table S1); 67 of these species were Actinopterygii, and eight were Elasmobranchii. Species were clumped into 61 genera, 36 families, and eight orders, of which Perciformes included 98.54% of the species. The most represented families were Pomacentridae, with six species, and Haemulidae, Scaridae, and Serranidae, with five species each. The species *Prionurus laticlavius* (Valenciennes, 1846) (17.9%), *Thalassoma lucasanum* (Gill, 1862) (13.1%) and *Halichoeres dispilus* (Günther, 1864) (11.1%) constituted the highest share of the whole counted fishes.

The sea temperatures recorded at the sampling sites are shown in Table 1. The temperature patterns were similar among sites and were significantly higher during the warm season (Wilcoxon paired test, P > 0.05 in all comparisons; Supplementary Fig. S1 and Supplementary Table S2). The average (minimum, maximum) temperature during the warm season was 26.8 °C (20–30.5 °C) and that during the cold season was 20.5 °C (16.6–25 °C).

**Figure 2:**
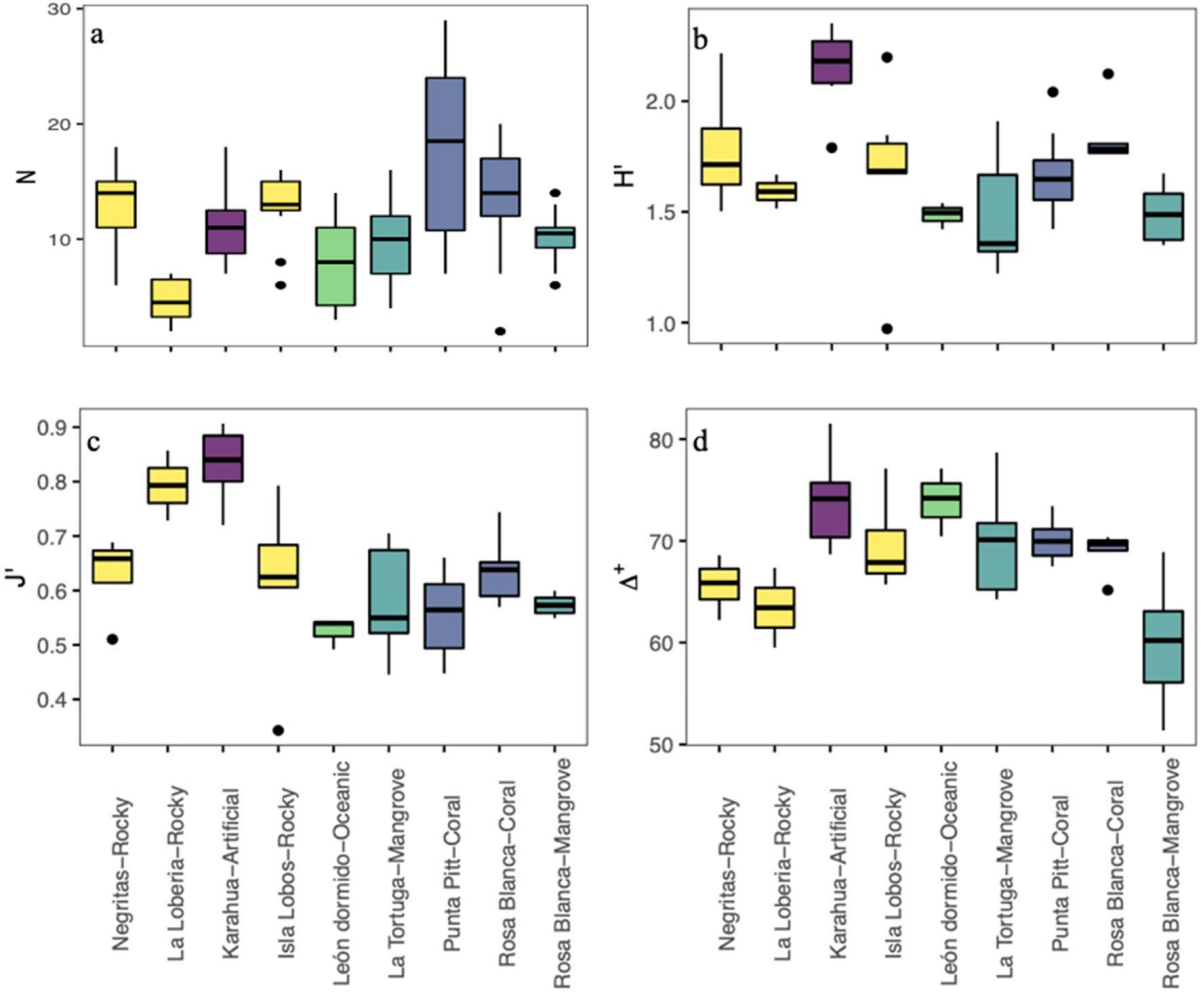
Fish community indices in the different coastal habitat types (sites/substrates) in the southeastern Galapagos Islands: (**a**) species richness (N), (**b**) Shannon index (H′), (**c**) Pielou’s evenness (J′), and (**d**) average taxonomic distinctness (Δ+).

The species richness mean values (± standard deviation) were higher in the warm season (25.6 ± 9) than in the cold season (22.3 ± 7.67), although they did not differ statistically (Kruskal–Wallis test, $\chi$^2^ = 0.259, df = 1, P = 0.610); Supplementary Fig. S2 and Supplementary Table S2). However, we found differences among sites (Kruskal–Wallis test, $\chi$^2^ = 29.74, df = 8, P = 0.0001; Fig. 2a). Punta Pitt and Rosa Blanca, which had the presence of coral substrate, had the highest numbers of species during both seasons, while La Lobería, which had rocky substrate, had the lowest species richness in both seasons. Other sites with rocky substrates, such as Negritas and Isla Lobos, had intermediate levels of species richness in both seasons, which were slightly higher than those in La Tortuga and Rosa Blanca, which had mangrove substrates. León Dormido, the oceanic habitat, had lower species richness than La Tortuga and Rosa Blanca (mangrove). Karahua, which had artificial substrate, had a low number of species during the warm season, but the number of species was high during the cold season. The most abundant species at each site are shown in Table 2. In general, *Stegastes beebei* (Nichols, 1924), *H. dispilus* and *T. lucasanum* were among the most abundant fish species in rocky habitats; *Paranthias colonus* (Valenciennes, 1846), in oceanic habitat; *Stegastes arcifrons* (Heller & Snodgrass, 1903), in mangrove habitat; and *P. laticlavius* and *T. lucasanum*, in coral and artificial habitats.

**Table 2:** Relative abundance (%) of most abundant fish species at each sampling site.

Punta Pitt-Coral	Rosa Blanca-Coral	Karahua-Artificial
*Prionurus laticlavius* (39.74%)	*Halichoeres dispilus* (27.0%)	*Thalassoma lucasanum* (17.01%)
*Apogon atradorsatus* Heller & Snodgrass, 1903 (18.46%)	*Thalassoma lucasanum* (23.61%)	*Prionurus laticlavius* (16.46%)
		*Stegastes arcifrons* (15.25%)

Isla Lobos-Rocky	Negritas-Rocky	La Lobería-Rocky
*Stegastes beebei* (21.72%)	*Halichoeres dispilus* (32.46%)	*Thalassoma lucasanum* (22.09)
*Xenocys jessiae* Jordan & Bollman, 1890 (16.35%)	*Prionurus laticlavius* (17.27%)	*Abudefduf troschelii* (Gill, 1862) (20.60%)
*Halichoeres dispilus* (15.22%)	*Stegastes beebei* (16.46%)	*Stegastes arcifrons* (19.40%)

Rosa Blanca-Mangrove	La Tortuga-Mangrove	León Dormido-Oceanic
*Stegastes arcifrons* (28.66%)	*Stegastes arcifrons* (52.55%)	*Paranthias colonus* (60.30%)
*Scarus ghobban* Forsskål, 1775 (28.27%)		
*Thalassoma lucasanum* (27.95%)		

The ecological diversity of the fish community did not show significant differences between seasons by site (Wilcoxon paired test, P > 0.05 in all comparisons; Supplementary Fig. S3 and Supplementary Table S2). In most cases, the variation during each season was low, except for the cold season in Isla Lobos and La Tortuga. The mean diversity values per site ranged from 1.3 to 2 decits/ind in the warm season and 1.4 to 2.3 decits/ind in the cold season. However, differences were found among the sites/substrates analyzed (Kruskal–Wallis test, $\chi$^2^ = 23.51, df = 8, P = 0.0028; Fig. 2b). In general, the highest diversity values were in the artificial substrate (Karahua), while the lowest values were in the mangrove and oceanic substrates in both seasons (La Tortuga, Rosa Blanca (mangrove) and León Dormido). The evenness also did not show significant differences among seasons (Wilcoxon paired test, P > 0.05 in all comparisons; Supplementary Fig. S4 and Supplementary Table S2). The mean evenness values per site ranged from 0.5 to 0.8 in the warm season and 0.4 to 0.9 in the cold season. However, significant differences were found among substrates (Kruskal–Wallis test, $\chi$^2^ = 25.51, df = 8, P = 0.0013), with the highest evenness value being the artificial substrate and the lowest being the oceanic and mangrove substrates (León Dormido and Rosa Blanca (mangrove); Fig. 2c).

**Figure 3:**
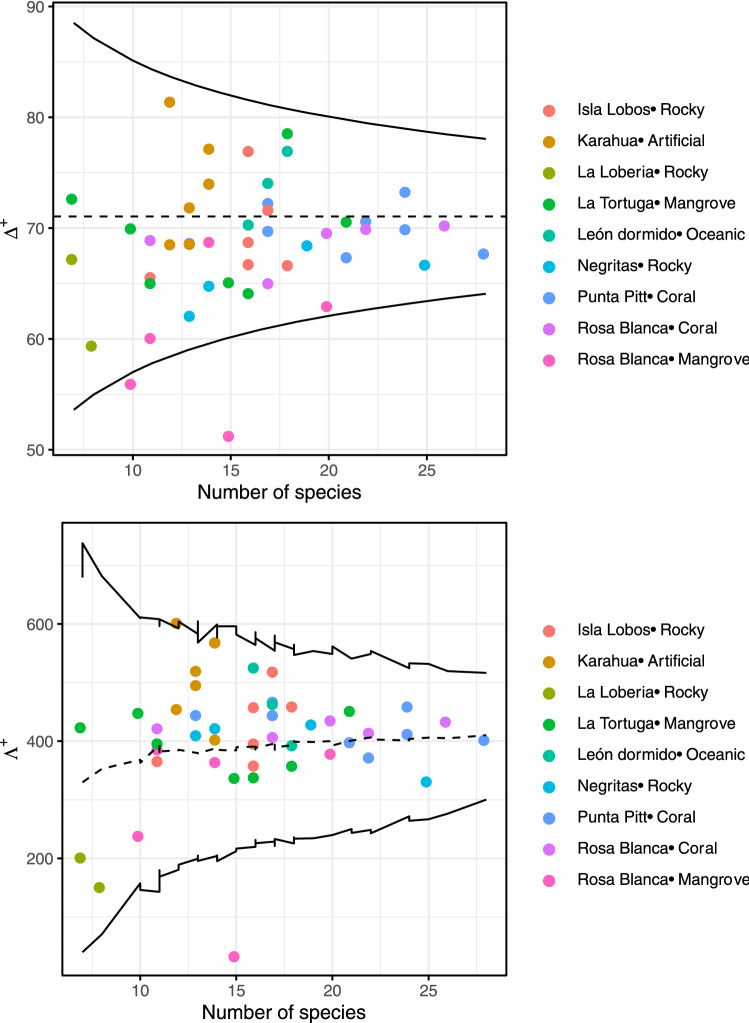
Funnel plot of the simulations of expected and observed average taxonomic distinctness (Δ+) and variation in taxonomic distinctness (Λ ) per site of the fish community in the southeastern Galapagos Islands. Each dot indicates each sample, and its color indicates site. The limits within thin lines indicate 95% of the simulated Δ+ and Λ+ values.

In relation to the average taxonomic distinctness, we also found no significant differences among seasons per site (Wilcoxon paired test, P > 0.05 in all comparisons; Supplementary Fig. S5 and Supplementary Table S2). The mean values per site ranged from 59 to 80 in the warm season and from 57 to 73 in the cold season. However, when comparing the values of average taxonomic distinctness among sites/substrates using a Kruskal–Wallis test, we found statistical differences ($\chi$^2^ = 22.4, df = 8, P = 0.0042; Fig. 2d). The greatest values were in the artificial substrate (Karahua), and the lowest were in the mangrove substrate [Rosa Blanca (mangrove)]. The comparison of average taxonomic distinctness observed and expected values is displayed in Fig. 3. We found that for all sites, the values were within the 95% probability limits of the simulation, meaning that the observed values were not significantly different from the expected value, except for one mangrove sampling point at the Rosa Blanca site. Additionally, the variation in the average taxonomic distinctness observed values were not different from the expected values, with higher variation and a lower number of species. The values and the variation in the taxonomic distinctness seemed to be very heterogeneous among sites, indicating similarities in the taxonomic composition of the fish community among sites.

**Figure 4:**
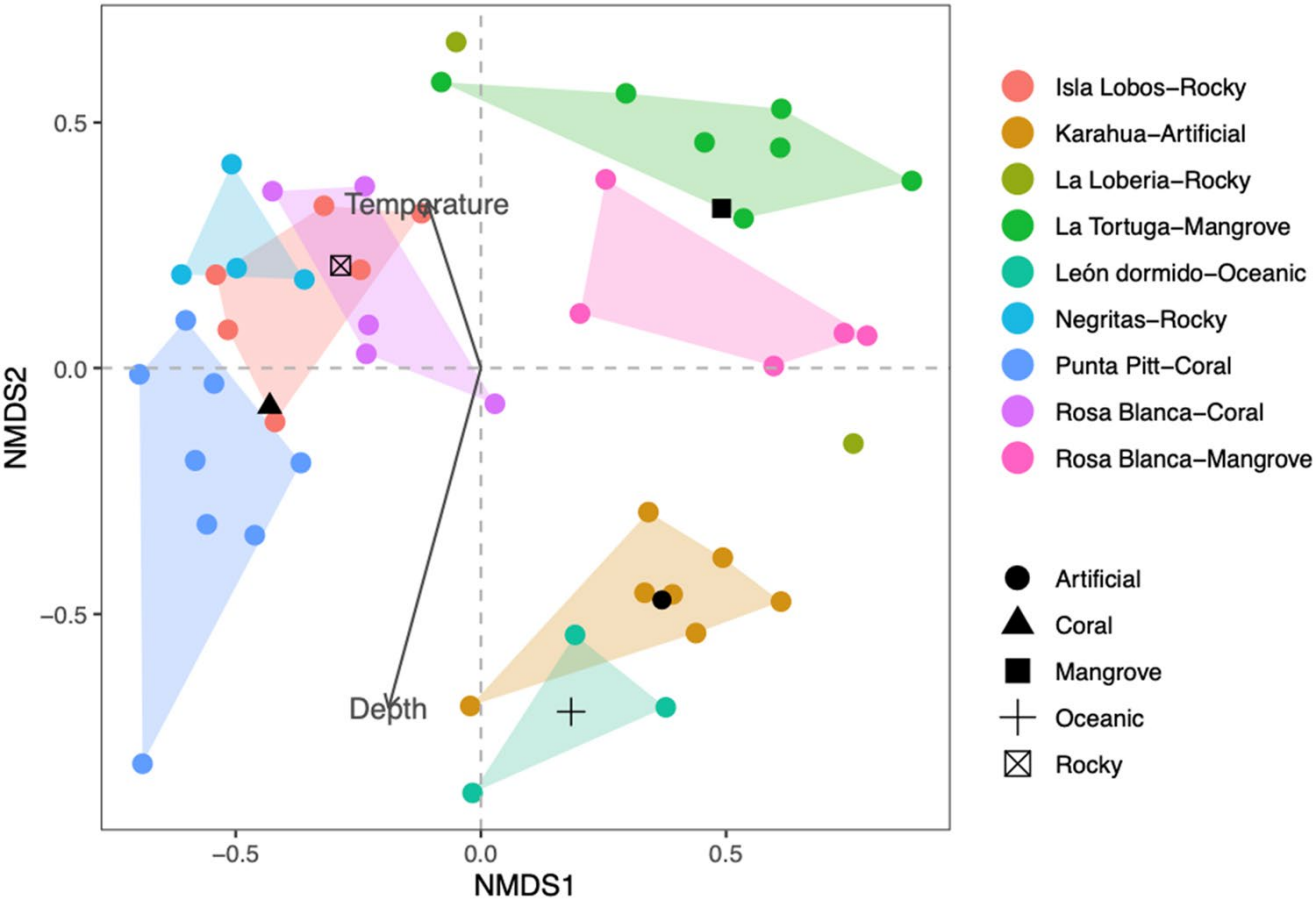
Non-metric multidimensional ordination based on the abundance per sample (dots) of the fish community in the southeastern Galapagos Islands. Convex hulls encloses all samples per site. Additionally, the vectors and centroids of the environmental variables correlate with the fish community structure.

The ecological and taxonomic diversity results contrasted with those obtained by the non-metric multidimensional scaling analysis based on the species composition similarity matrix among sites, which showed an acceptable ordering of spatial variation for several sites with a stress value of 0.2 (Fig. 4). Although stress values lower than 0.1 are considered to yield good ordinations results, according to^49^, a stress level of 0.2 could still lead to a usable interpretation in ecological data. The analysis showed that the different groups were mainly related to substrate type. We identified three groups: one included the sampling sites from oceanic and artificial substrates, the second integrated the mangrove areas, and the third included sites from the rocky and coral substrates. The ANOSIM of the species composition between the areas resulted in significant differences (R = 0.714, P = 0.001). The BIOENV test results demonstrated that the substrate had the strongest correlation with the fish community structure (r_s_ = 0.42), followed by the combination of substrate and depth (r_s_ = 0.40), and the combination of substrate, depth and temperature was the least correlated (r_s_ = 0.36). In Fig. 4, we show how the oceanic and artificial sampling sites were strongly associated with depth and negatively associated with temperature. Conversely, the coral, rocky and mangrove sites were more associated with temperature. From the MDS test results, we identified the fish species contributing more to the dissimilarity between the oceanic-artificial, coral-rocky, and mangrove groups of sampling sites. The average between-group dissimilarities and the species contributing more to these differences are presented in Table 3. In general, all comparisons showed high values of dissimilarity, at approximately 80%, indicating that the fish community abundance was different between groups. However, species such as *P. laticlavius, T. lucasanum,* and *Apogon atradorsatus* were more influential in all comparisons, indicating that the specific abundance of those species was very different among sites.

**Table 3:** SIMPER results showing the average between-group dissimilarities and species that contributed more to those dissimilarities.

Comparison	Average dissimilarity (%)	Species	Cumulative contribution (%)
Coral-Rocky vs Mangrove	88	*Stegastes arcifrons*	15
		*Prionurus laticlavius*	30
		*Halichoeres dispilus*	42
		*Thalassoma lucasanum*	52
		*Stegastes beebei*	61
		*Scarus ghobban*	68
		*Apogon atradorsatus*	73
Coral-Rocky vs Oceanic-Artificial	83	*Prionurus laticlavius*	16
		*Halichoeres dispilus*	29
		*Paranthias colonus*	42
		*Stegastes beebei*	53
		*Apogon atradorsatus*	62
		*Thalassoma lucasanum*	71
Mangrove vs Oceanic-Artificial	80	*Stegastes arcifrons*	21
		*Paranthias colonus*	36
		*Thalassoma lucasanum*	48
		*Scarus ghobban*	56
		*Apogon atradorsatus*	62
		*Prionurus laticlavius*	68
		*Eucinostomus dowii*	72

## Discussion

Coastal habitats include productive zones that provide important ecosystem services^26^ and essential fish habitats^50^. They are relevant grounds for feeding, nursery and spawning of several fish species with commercial and ecological value in the Galapagos archipelago^2,17,23–25^. The structural heterogeneity of the coastal habitat as well as the dynamics and exposure to ocean currents are important environmental factors that influence the diversity and abundance of fish species^2,10,51–54^. Fish move seasonally through different coastal habitats during their life cycle^22,26^, according to their environmental tolerance.

The distribution area of each species is influenced by the different evolutionary processes that have shaped organisms and, consequently, have determined their presence at certain sites^55^. The fish communities in the study area are composed of benthonic and demersal species, which are common in shallow waters with affinities to rocky, coral and sandy bottoms and have distinct zoogeographic affinities. Several of the most abundant species at each sampling site have an affinity for more than one substrate and are present in different types of habitats (i.e., coral, rocky, mangrove, oceanic and artificial), differing only in their percentage of abundance at each type. Some of these species (e.g., *Stegastes beebei, S. arcifrons, Thalassoma lucasanum* and *Prionurus laticlavius)* are of Indo-Pacific origin, and some are of wide distribution as *Halichoeres dispilus* and *Paranthias colonus*, which is characteristic in the southeastern of the archipelago^2,11^.

Fish communities in coral habitats are the most former, complex and diverse in the world, and their abundance and distribution are linked to the environmental traits and the feeding requirements of each species^56^. Our results indicate that the coral habitats have higher species richness and fish abundance, followed by the rocky and mangrove habitats. Both the coral and rocky habitats offer a variety of areas for refuge, feeding, nurturing and reproduction for many fish species^8^, and rocky habitats are particularly extensive on the Galapagos shelf^2,18,43^. In the same way, mangroves present highly variable physical conditions and provide better essential fish habitat, especially for juvenile blacktip sharks^25^; however, the species richness is lower than in other coastal habitats and comparable to that reported in mangrove bays on Santa Cruz Island in the center of the archipelago^24^.

Coastal habitats, especially reefs, are greatly diverse, and dominant species are influenced by the environmental variability, affecting the spatial and temporal heterogeneity of the communities^26^. The habitat seasonality, composition and richness, and responses of organisms may be affected by hydrological processes^57,58^. We found similar diversity of fish species throughout the year in coastal habitats on San Cristóbal Island, and the diversity seems not to be influenced by seasonality. Both the ecological and taxonomic diversity indices applied here are consistent in these results, suggesting seasonal environmental variability might not significantly influence the temporal and spatial heterogeneity of fish communities. The isolation of the Galapagos archipelago and its location in a convergence zone provides it unique oceanographic conditions^1–3^ that determine the specific species composition and biodiversity between regions^2,39,59^. Coastal habitats fish assemblages are influenced by specific thermal characteristics of each region within an archipelago^59^, as reported, in mangrove and rocky reef habitats in the Galapagos and the Solitary Islands, Australia^2,25,39,60^. Thus, species living around the islands in the same bioregion are adapted to the climatic heterogeneity that might influence a low species turnover between seasons.

Habitat characteristics as depth, heterogeneity (number of cavities), or structural complexity (diversity of substrate types or communities) determine the specific richness between localities on the same island^59^, as reported in fish communities in the Mediterranean or the Galapagos^39,61^. The proximity of sampling sites might contribute to the lack of significant differences in fish assemblages among coastal habitats type. The distance between nearby sampling sites in this study ranged from 1 to 21 km (10 km apart on average), with the closest locations being Rosa Blanca-coral and Rosa Blanca-mangrove. Strong connectivity in mangrove and reef habitats has been reported in the Galapagos, the Caribbean, and Indo-Pacific regions^25,62,63^. However, some authors^64,65^ have indicated that coral reef fish assemblages are usually not much influenced by nearby assemblages such as soft-bottom or mangrove-fish communities, and rather, seasonal variations are attributed to fish recruitment^66^.

The artificial habitat followed by coral habitats have the highest diversity values, and the diversity tends to be slightly higher in the cold season, although this is not similar at all sites. For example, in Las Negritas (rocky), Punta Pitt (coral), Rosa Blanca (coral) and Rosa Blanca (mangrove), diversity is slightly higher in the warm season, suggesting that new species visit these sites for feeding or reproduction. This is the case for snappers, *Lutjanus argentiventris* (Peters, 1869) and *L. viridis* (Valenciennes, 1846), which are only sighted during the warm season. This period coincides with their breeding season from April to May, as reported by^67^ on the Pacific coast of Mexico. Another example is *P. laticlavius* which is twice as abundant during warmer temperatures that is related to higher reproductive activity^68^.

Both environmental and anthropogenic factors influence the fish distribution, abundance, and diversity^33,40^. All sampling sites are affected by anthropogenic influences due to fishing and tourist grounds on this region. Although Galapagos artisanal fisheries are regulated by specific strategies to ensure the sustainability of the target species and reduce negative effects on the abundance of other species, it cannot be ruled out that this activity may affect the species diversity in each habitat^69,70^. In relation to tourism activities, all are regulated in the visit sites according to the Galapagos Management Plan and include, among others, a maximum number of visitors and certain allowed activities per site. Karahua is the only nonfishing site in this study and has the highest diversity and evenness, suggesting that it may be less impacted than other coastal habitats. However, this site corresponds to an artificial habitat for diving tourism and has different topographic characteristics from the other sites, which could influence the diversity of species and therefore was not comparable. Since fishing sites were not sampled for each coastal habitat in this study, it was not possible to accurately determine fishing effects on the richness and diversity of fish communities.

Higher structural complexity habitats are connected with higher diversity and fish abundances in marine waters^26,39,71,72^. For instance, on the western coast of Sweden and in the Mediterranean Sea, mussel beds and vegetated areas have high fish diversity^73–76^. On rocky reefs in the Mexican Pacific, rock and coral cover are highly related to dominant fish species and great diversity, while sandy bottoms have less influence on fish diversity^77^. On rocky habitats in the Galapagos Islands, no geographic patterns have been observed to explain the variability of fish abundance, richness, or diversity concerning the structural complexity of the habitat^39^. However, localities with higher roughness and number of cavities linked to a major structural complexity tend to present higher species richness^39^. We find slightly lower diversity values in mangrove habitats than in the other coastal habitats, suggesting geological structures are more important than vegetation in creating the structural complexity in the environments of the Galapagos Islands.

The poor water clarity (higher turbidity) in mangrove habitats may explain the low diversity, since the higher the turbidity, the lower the richness and diversity of fish species^78^. Turbidity modifies penetration and scattering of light^79^, influencing the foraging ability of visual hunting predators^80^. Although turbidity was not measured in this study, and we do not provide any data about it, it is realized that high turbidity may give predation shelter for benthic organisms and small juvenile fish^23,78^. Moreover, low visibility may influence the researchers’ ability to detect fish during the samplings, especially when using stereo-Baited Remote Underwater Video stations^25^. Although we had no difficulties in fish observation during the underwater visual surveys, we did not rule out that turbidity could affect the accuracy of the data obtained. Thus, further studies about these effects on mangrove fish communities are recommended.

It is important to consider some aspects that difficult the evaluation of fish communities and their relationship to particular habitats^26^. Fish frequently move between various environments^22^. The number and composition of fish species range with the hour of the day, being half to two-thirds of the species in most fish assemblages of diurnal habits^80,81^. Our visual censuses were developed during the same period of the day recording diurnal species. However, to reduce bias in the evaluation of diversity derived from sampling effort, we used a taxonomic diversity index, which is more susceptible to natural environmental variability and less sensitive to sample size variations^30^.

Lower values of taxonomic distinctness (Δ+) indicate more greatly impacted zones^82,83^. In this study, the Δ+ values were significantly different among sites, and Rosa Blanca (mangrove) presented the lowest values, indicating that the gatherings of closely related species made up the fish community. This may reduce the ichthyofauna responsiveness to stress factors in the ecosystem^57^, and therefore this area could be more impacted in relation to the other sites. Despite this result and the low diversity and evenness in Rosa Blanca (mangrove), this is one of the sites with higher fish abundance, mainly omnivorous (e.g., *S. arcifrons*) and herbivorous (e.g., *Scarus ghoban*) fishes which reflects a healthy habitat. This high abundance may be associated with fish mobility via superficial open ocean areas^22^ and the connectivity between adjacent habitats as coral reefs that may affect fish assemblages^84^.

Changes in community structure may be little perceptible for detecting in demersal communities^85^. Temporal or spatial community changes are susceptible to the sampling effort, thus multivariate techniques are recommended for evaluating the consistency of this group^34^. According to the MDS and ANOSIM tests, the fish community abundance may be distinct per group of habitats. These groups (oceanic and artificial; mangrove; coral and rocky) are identified by the type of substrate, while the composition of species in each group shows a distinct association strength with environmental variables, like substrate type, depth, and sea temperature.

The species composition in the oceanic and artificial habitats has a positive association with the depth and negative with the sea temperature. In contrast, the species composition in the coral, rocky and mangrove sites are more associated with sea temperature. This trend is related to the ecological traits of fish species but also to the availability resources, since certain fish species with certain set of traits are more associated with some habitats than others expressing their habitat preferences^34,72^. For example, the Pacific creole-fish (*Paranthias colonus*), which is the most abundant species in the oceanic habitat, has planktivorous habits that are usually found in deeper waters (from 10 to 70 m) than the other most abundant species in the rest of the sites. The deepest dives were made in oceanic and artificial habitats in 2011, which was a cooler year than 2010. These factors may have favored the abundance of food in these habitats and therefore in a greater abundance of species. On the other hand, species that inhabit shallow, warm waters and graze on algae and invertebrates (e.g., *S. beebei, S. arcifrons, T. lucasanum*, *P. laticlavius, H. dispilus, Xenocys jessiae* and *Apogon atradorsatus*) are more abundant in the inshore habitats, such as coral, mangrove and rocky sites, which have high structural complexity.

The diversity patterns observed in this study seem to be most strongly related to the type of substrate. The BIOENV test also indicates that the combination of the substrate and depth influences the fish community structure, and the sea temperature has less influence. The habitat complexity and the diversity of sea bottom types are important factors influencing the abundance patterns of many marine species^39,71,72,86^. Depth has also been considered a significant variable influencing fish community structure^34,39,72^. Habitat structural complexity complements depth, mainly for those species less influenced by depth or where depth and habitat complexity interact to influence fish abundance^72^. Thus, according to the similarity analysis, certain spatial differences in community structure are mainly influenced by three species (i.e., *P. laticlavius, T. lucasanum* and *A. atradorsatus*) with very different abundances between the groups of habitats.

Despite the strong seasonality observed during the sampling years, with the average sea temperatures reaching differences of 6 °C between the warm and cold seasons, no temporal variation was detected in the diversity patterns and community structure of fish at any site. Several studies have demonstrated the influence of sea temperature on diversity patterns in fish communities^25,33,34,40,87^. However, our results show that fish communities in the coastal habitats within a region of the Galapagos Islands have a high environmental tolerance, which allows them to persist in their habitat despite drastic seasonal changes in sea temperature. But extreme thermal impacts (i.e., elevated temperatures prevailing for over 12 months) during strong El Niño events could affect fish communities due to the loss of coral and macroalgal beds^88,89^. The environmental tolerance, a product of evolutionary processes^55^, allows species to adapt to environmental variability and unpredictability in the productivity of the Galapagos archipelago^11^.

## Conclusion

We determined that substrate and depth influence the fish communities’ structure and diversity patterns in the coastal habitats in the southeastern Galapagos archipelago. The community structure differs spatially, and the mangrove habitats have lower diversity values. The diversity patterns are more associated with the substrate type and the habitat's depth and less influenced by the seasonal sea temperature, and fish communities show high environmental tolerance. Although this study did not determine the artisanal fishing influence on the structure of the fish community, fishing might regulate the abundance of species and affect diversity. Therefore, further studies are required to determine these effects in the coastal habitats of the Galapagos Islands.

## Supplementary Information

Supplementary Information.
