# Supplementary material for: Fish diversity patterns along coastal habitats of the southeastern Galapagos archipelago and their relationship with environmental variables
Source: Sci Rep. 2022 Mar 4;12:3604. doi: 10.1038/s41598-022-07601-w (PMC8897472; doi:10.1038/s41598-022-07601-w)

Electronic Supplementary Material

**Fish diversity patterns along coastal habitats of the southeastern Galapagos archipelago and their relationship with environmental variables**

*Marjorie Riofrío-Lazo^1^, Manuel J. Zetina-Rejón^2^, Leandro Vaca-Pita^3^, Juan Carlos Murillo-Posada^4^ and Diego Páez-Rosas^1,5^

^1^ Universidad San Francisco de Quito and Galapagos Science Center. Isla San Cristóbal, Galápagos, Ecuador.

^2^ Instituto Politécnico Nacional, Centro Interdisciplinario de Ciencias Marinas (CICIMAR-IPN), La Paz, Baja California Sur, México.

^3^ Guía Naturalista en Patrimonio Turístico del Parque Nacional Galápagos. Islas Galápagos, San Cristóbal, Ecuador.

^4^ Pontificia Universidad Católica del Ecuador - Sede Manabí, Facultad de Biología. Bahía de Caráquez, Ecuador.

^5^ Dirección del Parque Nacional Galápagos, Unidad Técnica Operativa San Cristóbal. Isla San Cristóbal, Galápagos, Ecuador.

*** - Corresponding author:**

Dr. Marjorie Riofrío-Lazo

Universidad San Francisco de Quito and Galapagos Science Center

Isla San Cristóbal, Galápagos, Ecuador

Email: [mriofriol@usfq.edu.ec](mailto:mriofriol@usfq.edu.ec)

February 2022

**Table S1.** Density (ind/100m^2^) of fish species recorded in the sampling sites representing five coastal habitats in San Cristóbal Island at the southeast of the Galapagos archipelago.

| Species | Punta Pitt-Coral | Rosa Blanca-Coral | Rosa Blanca-Mangrove | Negritas-Rocky | La Loberia-Rocky | Karahua-Artificial | Isla Lobos-Rocky | León Dormido-Oceanic | La Tortuga-Mangrove |
| --- | --- | --- | --- | --- | --- | --- | --- | --- | --- |
| *Abudefduf troschelii* | 36.00 | 1.83 | 66.50 | 4.44 | 23.00 | 41.25 | 6.68 | 25.08 | 46.78 |
| *Acanthemblemaria castroi* |  |  |  | 0.22 |  |  |  |  |  |
| *Acanthurus xanthopterus* | 10.83 | 1.40 |  |  |  |  |  |  |  |
| *Aetobatus narinari* |  |  | 0.25 |  |  | 0.25 |  | 0.25 |  |
| *Anisotremus interruptus* | 149.42 | 5.63 |  | 0.25 |  | 4.00 | 8.05 |  | 0.10 |
| *Apogon atradorsatus* | 550.50 | 70.00 |  |  |  | 59.25 | 46.40 | 62.83 |  |
| *Archosargus pourtalesii* |  |  |  |  |  | 14.25 |  | 13.17 | 1.67 |
| *Arothron meleagris* |  | 0.40 |  |  |  |  |  |  |  |
| *Aulostomus chinensis* | 0.67 | 0.20 |  |  |  |  | 0.33 |  |  |
| *Balistes polylepis* | 0.50 |  |  |  |  |  |  |  |  |
| *Bodianus diplotaenia* | 132.67 | 32.55 | 0.80 | 33.77 |  |  | 29.95 | 1.50 | 5.73 |
| *Canthigaster punctatissima* |  | 0.38 |  |  |  |  |  |  |  |
| *Caranx sexfasciatus* |  |  |  |  |  |  |  |  | 2.00 |
| *Carcharhinus falciformis* |  |  | 0.45 |  |  |  |  |  |  |
| *Carcharhinus galapagensis* |  |  |  |  |  |  |  | 1.50 |  |
| *Carcharhinus limbatus* |  |  |  |  |  | 0.50 |  | 3.42 |  |
| *Cephalopholis panamensis* | 3.08 | 0.65 |  | 0.33 |  |  | 0.42 |  | 0.33 |
| *Chaetodon humeralis* | 16.33 |  | 0.20 |  |  |  |  |  | 0.75 |
| *Chilomycterus reticulatus* |  |  |  |  |  | 0.50 |  | 1.75 |  |
| *Chromis atrilobata* | 1.33 | 1.00 |  | 15.78 |  |  | 40.55 | 60.42 | 34.00 |
| *Cirrhitus rivulatus* | 1.50 | 0.75 |  | 0.78 | 1.33 | 1.75 |  | 1.25 | 0.10 |
| *Dasyatis brevis* | 5.58 |  | 0.80 |  |  | 0.75 |  | 0.50 | 0.18 |
| *Diodon holocanthus* | 0.17 | 0.25 |  |  |  | 0.50 |  |  |  |
| *Diodon hystrix* |  |  | 0.20 |  |  | 0.25 |  | 0.42 | 0.14 |
| *Echidna nocturna* |  | 0.13 |  |  |  |  |  |  |  |
| *Epinephelus labriformis* | 5.83 | 0.50 |  | 1.88 |  | 2.00 | 2.33 |  | 0.18 |
| *Eucinostomus argenteus* |  |  |  |  |  | 8.00 |  | 6.00 |  |
| *Eucinostomus dowii* | 0.17 |  |  |  |  |  |  |  | 72.27 |
| *Fistularia commersonii* | 0.17 | 1.45 | 1.45 | 0.11 |  |  | 0.17 |  |  |
| *Girella freminvillii* | 1.42 |  |  |  |  |  |  |  |  |
| *Gobioclinus dendriticus* | 0.33 | 2.63 | 0.20 | 2.09 |  | 10.00 | 4.52 | 1.00 | 6.63 |
| *Haemulon scudderii* | 11.83 | 8.60 | 2.40 | 0.25 | 6.00 |  | 0.20 |  | 6.84 |
| *Halichoeres dispilus* | 181.75 | 309.93 | 11.20 | 271.67 | 12.33 |  | 239.20 |  | 18.24 |
| *Halichoeres nicholsi* | 8.42 | 13.10 | 1.00 | 6.19 | 1.33 | 1.00 | 2.15 | 0.25 | 3.86 |
| *Hippocampus ingens* |  |  | 0.20 |  |  |  |  |  |  |
| *Holacanthus passer* | 14.58 | 4.38 | 0.80 | 12.32 |  | 5.75 | 12.33 | 16.50 |  |
| *Johnrandallia nigrirostris* | 29.42 | 0.63 |  | 1.61 |  | 2.00 | 1.92 | 5.92 | 0.09 |
| *Kyphosus elegans* | 1.00 |  |  | 0.25 |  |  |  |  |  |
| *Lutjanus argentiventris* | 1.25 |  | 14.60 |  |  | 1.00 |  | 1.25 | 7.92 |
| *Lutjanus novemfasciatus* |  |  | 0.40 |  |  |  |  |  | 7.06 |
| *Lutjanus viridis* | 4.42 | 3.00 | 12.60 | 0.50 | 1.00 |  |  | 8.00 |  |
| *Microspathodon bairdii* |  |  |  | 4.00 |  |  |  |  |  |
| *Microspathodon dorsalis* |  | 0.60 | 0.20 | 4.32 |  |  | 0.58 |  |  |
| *Mugil cephalus* |  |  |  |  |  | 4.50 |  |  |  |
| *Mugil thoburni* |  |  |  |  |  |  |  |  | 23.75 |
| *Mulloidichthys dentatus* | 12.50 |  |  |  |  |  |  |  |  |
| *Muraena argus* |  | 0.13 |  |  |  |  |  |  |  |
| *Muraena lentiginosa* |  | 0.58 |  |  |  | 0.25 |  |  |  |
| *Mycteroperca olfax* | 2.50 | 0.85 | 1.30 |  |  |  | 1.58 |  | 1.51 |
| *Myrichthys tigrinus* | 0.17 |  |  |  |  |  |  |  |  |
| *Nicholsina denticulata* |  | 0.60 | 0.20 | 0.11 | 0.33 |  |  |  |  |
| *Ophioblennius steindachneri* | 0.92 |  |  | 0.25 |  |  |  |  | 1.29 |
| *Orthopristis forbesi* | 5.00 |  |  |  |  |  |  |  | 0.18 |
| *Paranthias colonus* | 105.50 | 51.63 |  | 17.56 |  | 14.50 | 172.18 | 403.33 | 0.67 |
| *Pareques perissa* |  |  |  | 0.11 |  |  |  |  |  |
| *Plagiotremus azaleus* | 13.42 | 5.13 | 1.40 | 12.46 |  |  | 5.85 |  | 2.11 |
| *Prionurus laticlavius* | 1184.92 | 56.73 | 2.40 | 144.52 |  | 81.75 | 225.30 |  |  |
| *Scarus compressus* | 4.50 |  |  |  |  |  | 0.17 |  |  |
| *Scarus ghobban* | 2.75 | 50.40 | 298.45 | 0.53 |  |  | 0.50 |  |  |
| *Scarus perrico* | 4.75 | 0.50 |  |  |  |  | 3.25 |  |  |
| *Scarus rubroviolaceus* | 0.75 |  |  | 0.11 |  | 5.00 | 0.83 | 1.25 |  |
| *Serranus psittacinus* | 0.33 | 6.65 | 0.65 |  | 0.67 | 6.00 |  | 2.00 |  |
| *Sphoeroides annulatus* | 1.50 | 5.85 | 12.50 |  |  | 21.75 | 1.00 | 2.75 | 4.73 |
| *Sphyraena idiastes* |  |  |  |  |  | 25.50 |  |  |  |
| *Sphyrna lewini* |  |  |  |  |  |  |  | 0.75 |  |
| *Stegastes arcifrons* | 7.67 | 36.25 | 302.55 | 23.03 | 21.67 | 75.75 | 38.55 | 21.92 | 320.95 |
| *Stegastes beebei* | 119.92 | 180.68 | 18.20 | 137.73 | 19.33 | 3.25 | 341.40 |  | 3.42 |
| *Sufflamen verres* | 12.17 | 0.90 |  | 0.44 |  |  | 0.33 |  |  |
| *Synodus lacertinus* | 0.50 |  | 1.40 | 0.20 |  |  | 0.58 |  | 0.14 |
| *Taeniurops meyeni* | 1.75 |  | 0.60 |  |  |  | 0.25 | 0.42 |  |
| *Thalassoma lucasanum* | 271.67 | 266.10 | 295.10 | 134.99 | 24.67 | 84.50 | 127.03 | 25.42 | 13.05 |
| *Triaenodon obesus* |  |  | 6.65 |  |  | 7.25 |  |  |  |
| *Xenichthys agassizii* | 16.67 |  |  |  |  | 13.75 |  |  | 1.82 |
| *Xenocys jessiae* | 40.00 | 4.00 |  | 4.00 |  |  | 257.00 |  | 22.22 |
| *Zanclus cornutus* | 3.00 |  |  |  |  |  |  |  |  |
| Total per site | 2982 | 1126.9 | 1055.65 | 836.81 | 111.67 | 496.75 | 1571.6 | 668.83 | 610.74 |

**Table S2.** Wilcoxon paired test results. Comparisons of sea temperature, species richness, ecological diversity, Pielou’s evenness, and taxonomic distinctness between seasons in the different coastal habitat types (sites/substrates) in the southeastern of the Galapagos Islands.

| **Site/substrate** | **Sea temperature (°C)** | |  | **Species richness (N)** | |  | **Ecological diversity (H’)** | |  | **Pielou’s evenness (J’)** | |  | **Taxonomic distinctness (Δ+)** | |
| --- | --- | --- | --- | --- | --- | --- | --- | --- | --- | --- | --- | --- | --- | --- |
|  | **W** | **p value** |  | **W** | **p value** |  | **W** | **p value** |  | **W** | **p value** |  | **W** | **p value** |
| Punta Pitt-Coral | 29714 | 2.20E-16 |  | 19 | 0.936 |  | 6 | 0.69 |  | 7 | 0.89 |  | 10 | 0.69 |
| Rosa Blanca-Coral | 16224 | 2.20E-16 |  | 19 | 0.942 |  | 3 | 1.00 |  | 4 | 0.80 |  | 0 | 0.20 |
| Rosa Blanca-Mangrove | 6880 | 2.20E-16 |  | 8 | 0.396 |  | 0 | 0.20 |  | 2 | 0.80 |  | 0 | 0.20 |
| Negritas-Rocky | 12474 | 2.20E-16 |  | 11.5 | 0.351 |  | 0 | 0.33 |  | 0 | 0.33 |  | 2 | 1.00 |
| La Lobería-Rocky | 192 | 2.35E-07 |  | 2.5 | 0.507 |  | 1 | 1.00 |  | 1 | 1.00 |  | 1 | 1.00 |
| Karahua-Artificial | 6400 | 2.20E-16 |  | 11.5 | 0.384 |  | 10 | 0.10 |  | 10 | 0.10 |  | 0 | 0.08 |
| Isla Lobos-Rocky | 12126 | 2.20E-16 |  | 6.5 | 0.131 |  | 3 | 0.70 |  | 6 | 0.70 |  | 6 | 0.70 |
| León Dormido-Oceanic | 1536 | 1.73E-14 |  | 6.5 | 0.282 |  | 1 | 1.00 |  | 1 | 1.00 |  | 0 | 0.67 |
| La Tortuga-Mangrove | 16498 | 2.20E-16 |  | 47 | 0.466 |  | 7 | 0.86 |  | 11 | 0.11 |  | 4 | 0.63 |

**Figure S1.** Differences in sea temperatures between seasons in each sampling site in the southeastern of the Galapagos Islands. No comparison was significant at P = 0.05. See Table S2 for Wilcoxon paired test results.


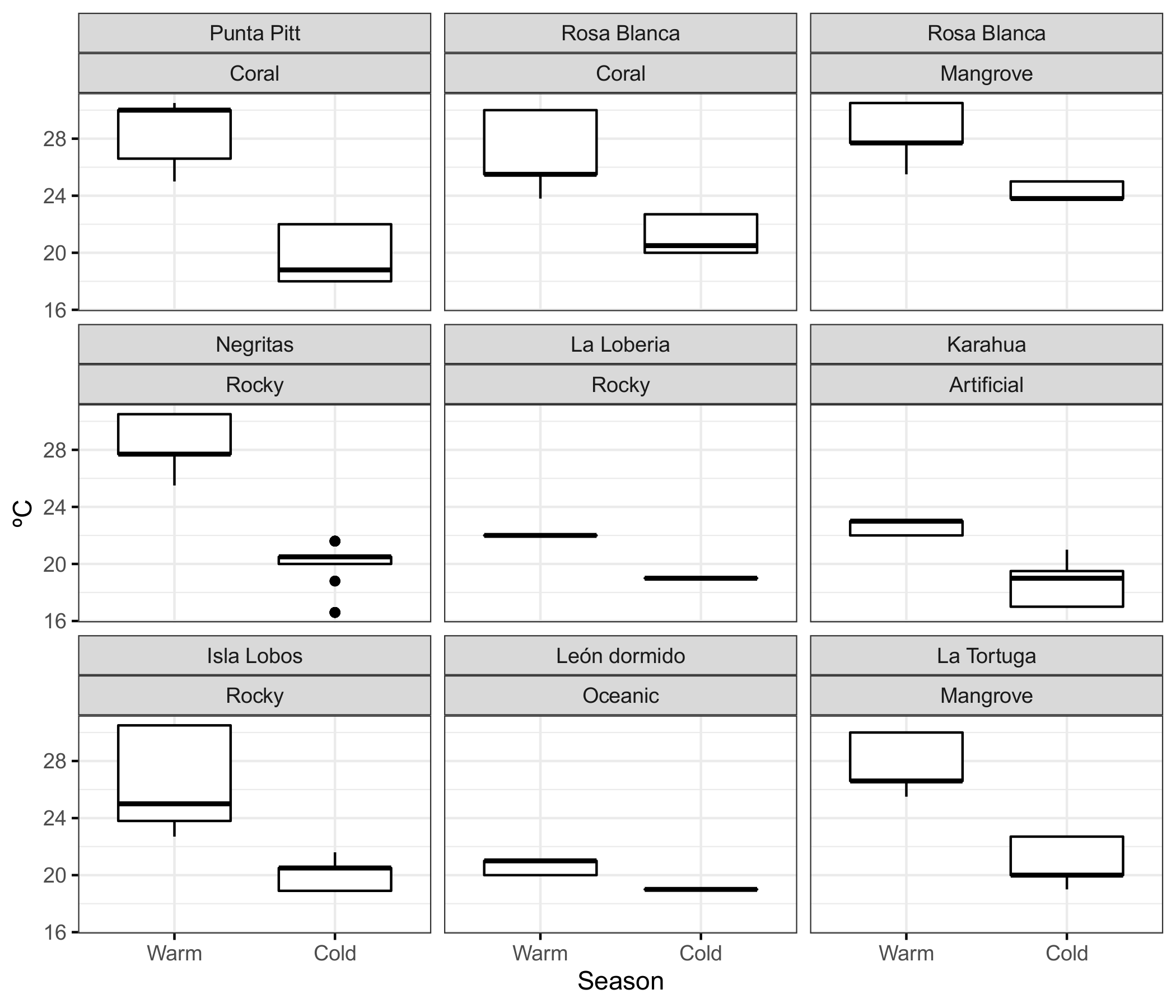


**Figure S2.** Species richness per site and season of the fish community in the southeastern of the Galapagos Islands. No comparison was significant at P = 0.05. See Table S2 for Wilcoxon paired test results.


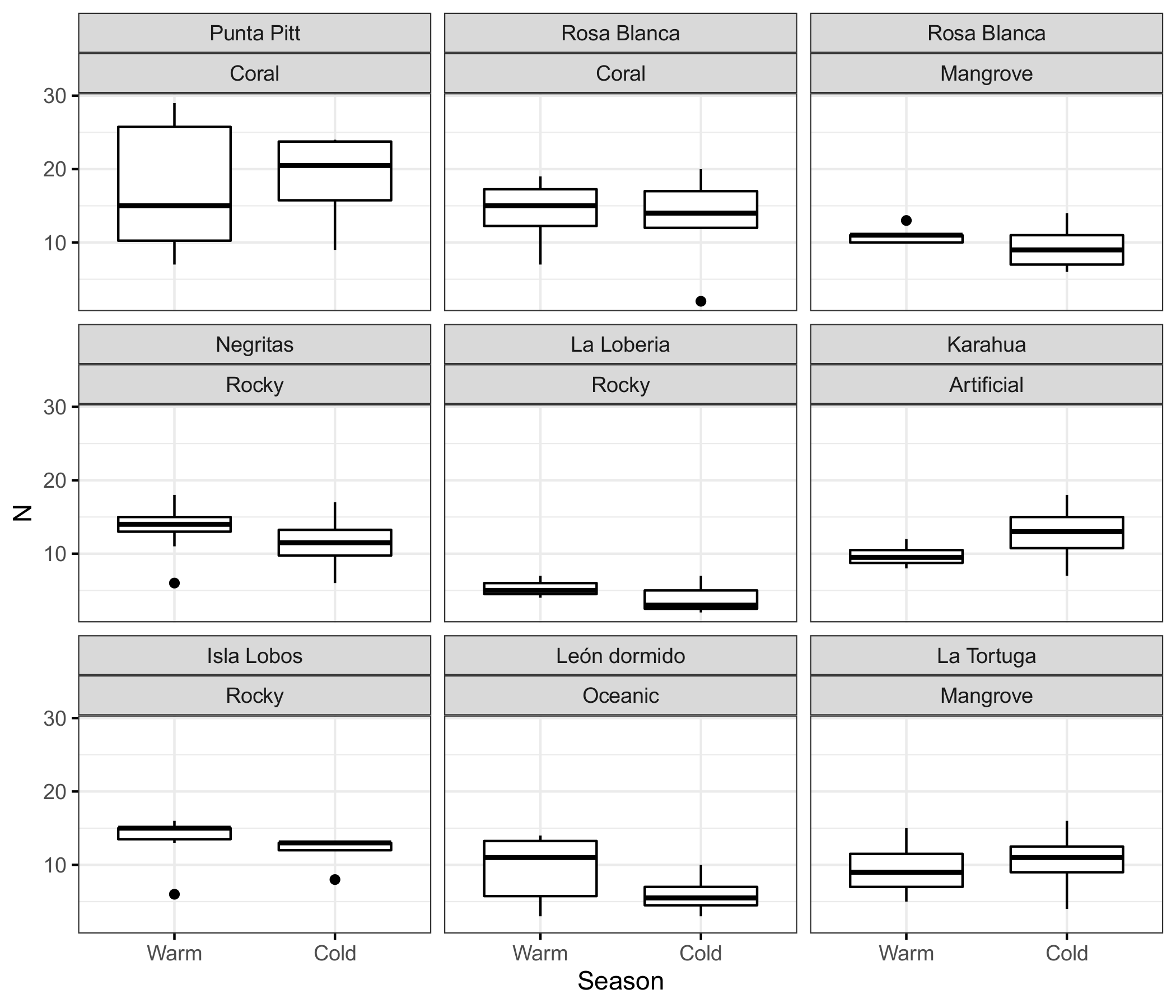


**Figure S3.** Ecological diversity per site and season of the fish community in the southeastern of the Galapagos Islands. No comparison was significant at P = 0.05. See Table S2 for Wilcoxon paired test results.


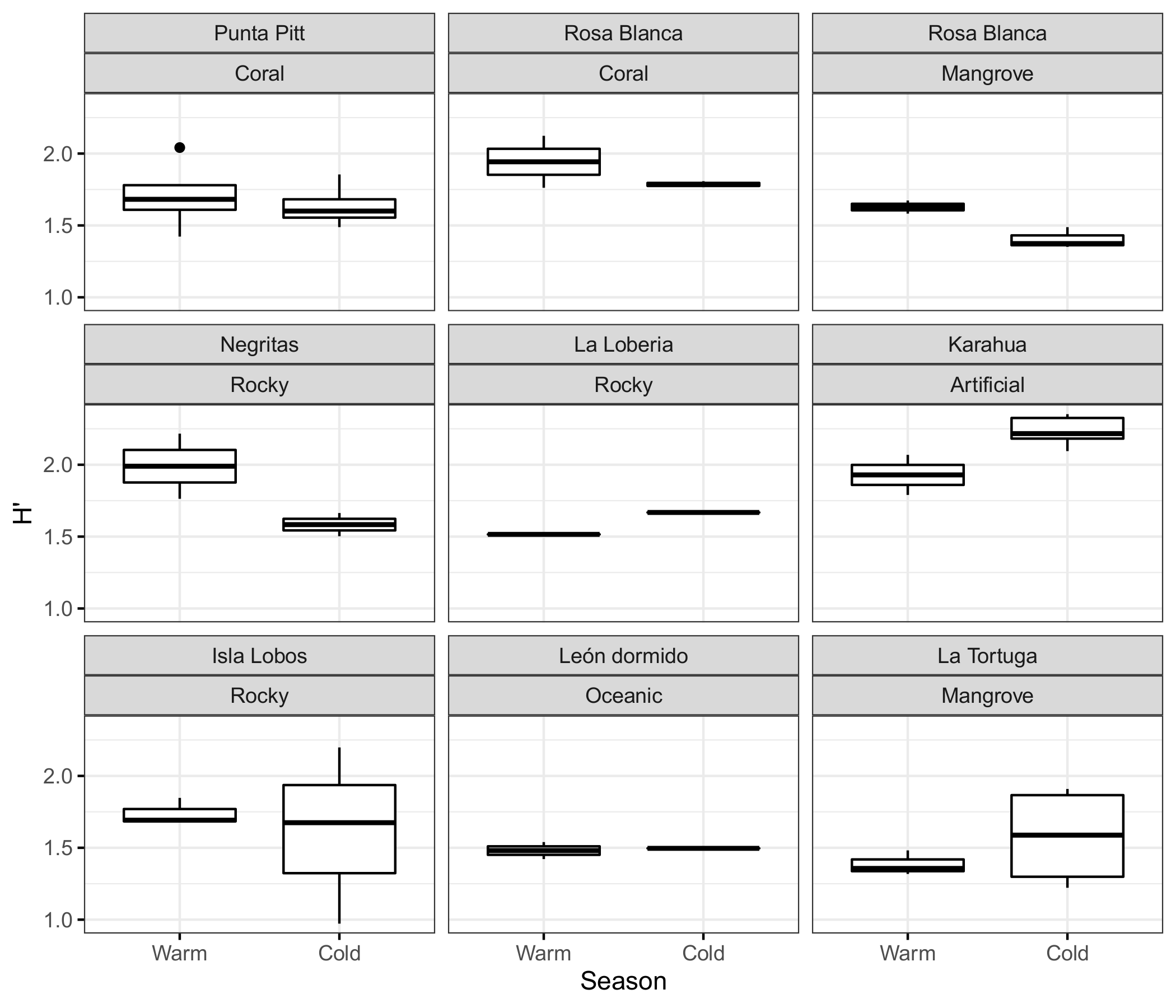


**Figure S4.** Pielou’s evenness per site and season of the fish community in the southeastern of the Galapagos Islands. No comparison was significant at P = 0.05. See Table S2 for Wilcoxon paired test results.


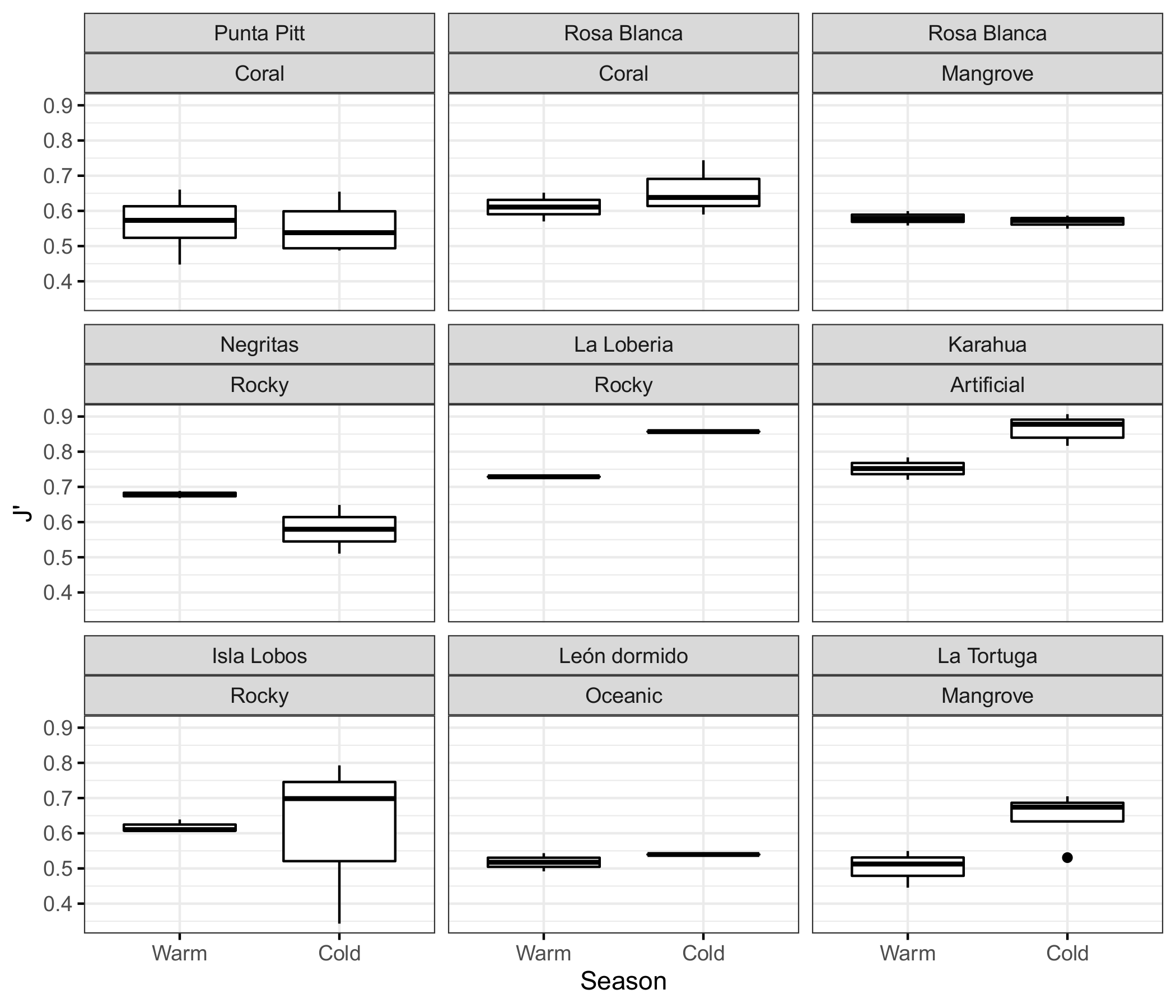


**Figure S5.** Taxonomic distinctness per site and season of the fish community in the southeastern of the Galapagos Islands. No comparison was significant at P = 0.05. See Table S2 for Wilcoxon paired test results.


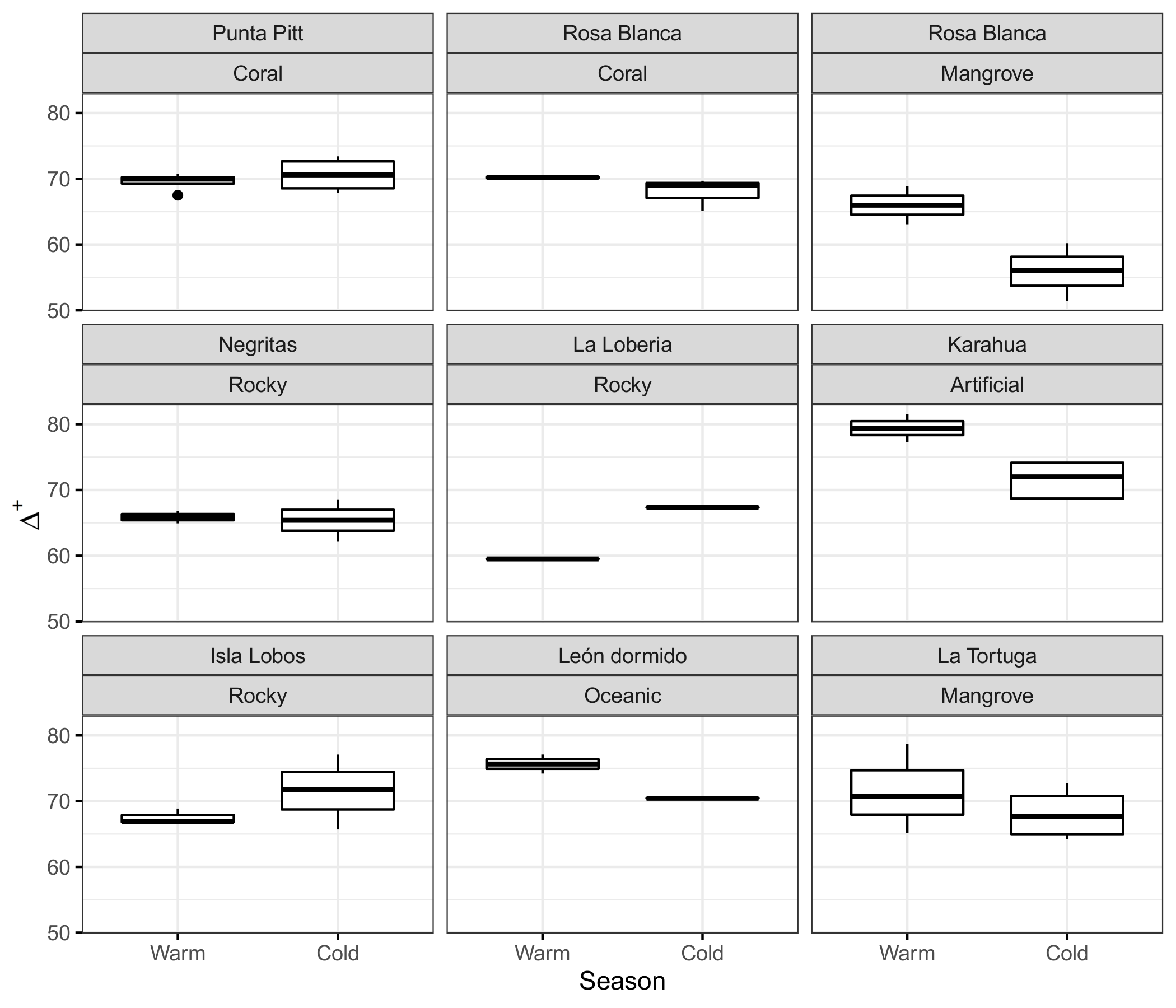

Supplement: Supplementary file 1 — Supplementary Information. [file 41598_2022_7601_MOESM1_ESM.docx]
